# Analytical Approaches for Detection, Characterization, and Environmental Significance of Suspended Particulate Matter in Aquatic Systems: A Scoping Review

**DOI:** 10.1155/sci5/6704648

**Published:** 2026-10-22

**Authors:** Pavlo Kuznietsov, Olha Biedunkova, Mykola Klymenko, Oleksandr Hrytsyna, Olha Varzhel, Maksym Stepanenko

**Affiliations:** ^1^ National University of Water and Environmental Engineering Soborna St. 11 Rivne 33028 Ukraine nuwm.edu.ua

**Keywords:** aquatic environmental monitoring, chemical characterization, contaminant transport, optical sensing, particle size distribution, suspended particulate matter, suspended solids, turbidity

## Abstract

Suspended particulate matter (SPM) represents a component of aquatic systems, acting both as a carrier of contaminants and as a factor shaping ecosystem functioning. This review summarizes recent progress in methods for detecting and quantifying SPM in natural waters. Traditional gravimetric analysis provides a direct mass‐based reference, while optical approaches such as turbidimetry and nephelometry enable rapid and automated assessments. Granulometric techniques, including sedimentation and laser diffraction, contribute to understanding particle size distribution and its role in transport, settling, and pollutant interactions. Chemical characterization, based on spectroscopic and spectrometric methods, identifies the composition of SPM and its association with heavy metals, organic pollutants, and microplastics. Particular attention is given to the ecological risks posed by fine particles, which remain suspended longer and enhance contaminant mobility. The review integrates findings from hydrochemistry, analytical chemistry, and environmental monitoring, outlining both the strengths and limitations of existing methods and highlighting directions for improving water‐quality assessment and ecological risk evaluation.

**Highlights**
• Systematizes analytical approaches for detection and characterization of SPM in aquatic systems.
• Compares gravimetric, optical, granulometric, and chemical techniques.
• Identifies calibration and standardization challenges limiting cross‐study comparability.
• Links particle size and composition with contaminant transport and ecological risk.
• Prioritizes real‐time monitoring, multimodal analysis, and microplastic integration.

## 1. Introduction

Suspended particulate matter (SPM) in an aquatic environment is a mixture of mineral, organic, or anthropogenic impurities that remain suspended in the water column [1]. The sources of such particles can be both natural processes, such as soil erosion, surface runoff, and biogenic activity, and anthropogenic activities, such as industrial emissions, agricultural runoff, wastewater discharge, and microplastic (MP) pollution. The presence of SPM affects water quality by reducing transparency, disrupting the photosynthetic activity of aquatic flora and fauna, promoting the spread of pathogens and toxic substances, and complicating the operation of water‐treatment systems [2]. That is why monitoring SPM is important for assessing the state of aquatic ecosystems, controlling drinking‐water quality, managing natural resources, and preventing environmental degradation [3]. In this review, SPM is used as the general term for particulate material maintained in the water column. Total suspended solids (TSS) refer specifically to the gravimetrically determined mass concentration of retained suspended material. The terms suspended solids and suspended sediments are used only when referring to the terminology employed in the cited studies or to specific sedimentological contexts.

Given the rapid expansion of analytical approaches applied to SPM in aquatic environments, a structured scoping review was conducted in accordance with the Preferred Reporting Items for Systematic Reviews and Meta‐Analyses extension for Scoping Reviews (PRISMA‐ScR). Scopus was selected as the primary bibliographic database because of its broad multidisciplinary coverage of environmental science, analytical chemistry, hydrology, aquatic ecology, and environmental monitoring, which corresponded to the interdisciplinary scope of this review. The use of a single database was intended to maintain a transparent and reproducible search strategy and to avoid combining records generated under substantially different indexing and retrieval algorithms. Web of Science and Google Scholar were not included because the objective was not to perform an exhaustive database‐wide systematic review, but to develop a structured scoping evidence base centered on analytical approaches to SPM in aquatic systems. Google Scholar was additionally not considered suitable as a primary source because of its less transparent indexing and reproducibility of search results. No language restriction was applied during the initial search; eligibility was determined according to the predefined relevance criteria. The literature search was performed in the Scopus database on May 15, 2026, and covered publications indexed from 1971 to the search date. Searches were conducted within titles, abstracts, and author keywords to identify studies in which SPM represented a substantive research topic rather than a secondary observation. The search strategy was designed to capture the principal methodological and environmental dimensions of SPM research, including SPM and suspended‐solids determination, turbidity and optical monitoring, particle‐size characterization, elemental and chemical analysis, MP identification, and studies addressing contaminant transport, ecological effects, and environmental significance. A total of 34,386 records were identified in Scopus across the predefined search domains. After removal of duplicate records, 23,812 records remained for title and abstract screening. At this stage, 21,134 records were excluded because they were not related to SPM, concerned nonaquatic or otherwise irrelevant contexts, were not eligible publication types, or did not address relevant analytical methods or outcomes. A total of 2678 reports were subsequently assessed for eligibility at the full‐text stage. Of these, 1987 reports were excluded because they did not report original data, were not focused on SPM, addressed irrelevant outcomes, or provided insufficient methodological detail. Ultimately, 691 studies met the predefined eligibility criteria and were included in the scoping review. The included evidence was subsequently organized into four thematic categories: (i) concentration and monitoring methods, (ii) particle‐size characterization, (iii) chemical composition and contaminant analysis, and (iv) environmental significance of SPM. This classification enabled a structured comparison of the analytical approaches used to quantify, characterize, and interpret SPM in aquatic systems, while also identifying methodological limitations affecting the comparability and environmental interpretation of reported results. The complete process of study identification, duplicate removal, screening, full‐text eligibility assessment, and inclusion is presented in the PRISMA‐ScR flow diagram (Figure 1). The resulting evidence base was used for the subsequent critical synthesis of SPM analytical methodologies and their applicability to water‐quality assessment, contaminant monitoring, and ecological risk evaluation.

**FIGURE 1:**
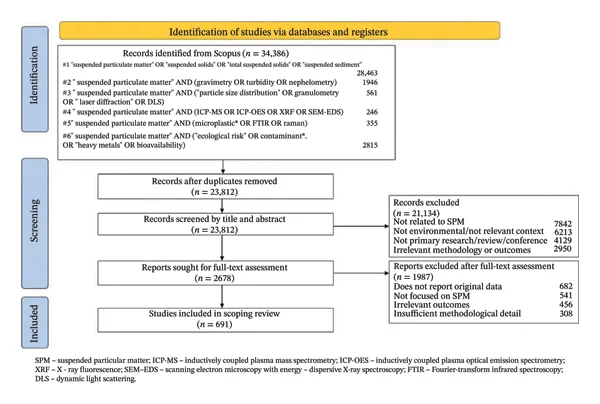
PRISMA‐ScR flow diagram illustrating the identification, screening, eligibility assessment, and inclusion of literature sources used in the scoping review of analytical approaches for SPM in aquatic systems.

Methods for detecting and quantifying SPM are based on the principles of gravimetry, optics, spectroscopy, microscopy, and physical separation (Figure 2a). Analytical methods include the traditional gravimetric approach—filtering and weighing the sediment—which is a fundamental tool [4]. Optical methods, such as turbidimetry, provide a quick indirect assessment of turbidity, but their effectiveness is limited by the dependence of the signal on particle composition [5]. Finally, sedimentation and centrifugation methods separate SPM from water for further analysis. The size of SPM determines the physical properties of particles, in particular, their diameter, shape, and aggregate state, which significantly affect transport processes in the aquatic environment, the bioavailability of sorbed pollutants, the rate of precipitation, and the efficiency of purification mechanisms [6]. Finely dispersed SPM has a larger specific surface area, which promotes more active sorption of toxic substances, in particular, heavy metals (HMs) and organic pollutants (OPs), and can remain in suspension for a long time, increasing the risk of their transport over long distances [7]. Such particles often penetrate filtration barriers, reducing the effectiveness of traditional water purification systems, and are potentially accessible to living organisms, causing negative biological effects [8]. On the other hand, larger SPM, although less mobile, often participates in the formation of bottom sediments, which act as secondary sources of pollution [9]. For this reason, the distribution of SPM by size fractions is a parameter in environmental monitoring, sedimentation process modeling, and the development of engineering solutions for water treatment. Equally important is the analysis of the chemical composition of SPM, which allows identifying sources of pollution, assessing the degree of ecological threat, and detecting potentially hazardous substances transported together with the solid phase [10]. Many studies [11, 12] confirm that it is the solid phase that is the main carrier of HM and OP, which can desorb from the surface of particles when the physical and chemical conditions of the aquatic environment change.

**FIGURE 2:**
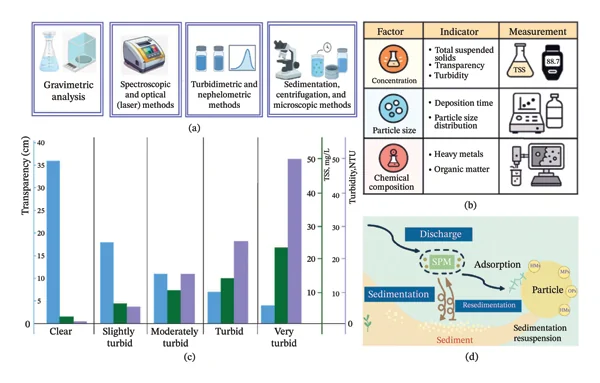
Methods for detecting and quantifying SPM (a); factors influencing SPM (b); еxamples of regulatory and ecological thresholds for suspended solids and turbidity (c); phenomena of transformation of SPM pollutant transport (d).

Although analytical technologies for the study of SPM have advanced considerably, several problems remain unresolved. A major limitation is the lack of standardized procedures, which complicates comparisons of data obtained in different regions and by different laboratories. Traditional methods often suffer from low sensitivity, variable results when water composition changes, high labor demands, and limited suitability for automated or real‐time monitoring. This review aims to systematize current methods for detecting and quantifying SPM in aquatic environments, evaluating their sensitivity, accuracy, strengths, and drawbacks. It also considers the prospects for further methodological development in the context of environmental monitoring and water resource management and integrates findings from hydrochemistry, analytical chemistry, and monitoring studies, providing an up‐to‐date assessment of the effectiveness of existing approaches and the areas where improvement is most needed.

## 2. Factors Influencing SPM

The impact of SPM on aquatic ecosystems is multidimensional and is determined not only by SPM concentration, but also by a number of other parameters, including particle size distribution (PSD), chemical nature, and stability in the aquatic environment (Figure 2b). Each of these factors has a specific mechanism of action, affecting both the physical characteristics of water and the physiological and biochemical processes in aquatic organisms.

### 2.1. SPM Concentration

Total SPM concentration is a commonly used indicator of suspended particulate pollution. It is traditionally determined gravimetrically by filtration, drying, and weighing of the retained suspended material to obtain the TSS concentration. Alternatively, optical (turbidimetric and nephelometric) methods are used, which provide an indirect assessment based on turbidity or transparency. Standardization of SPM concentration (Figure 2c) in water using different measurement methods demonstrates a consistent relationship between transparency, turbidity, and TSS indicators [13, 14]. Even in the short term, increased SPM concentrations can significantly reduce water transparency, hindering phytoplankton photosynthesis and reducing the primary production of the water body. From an ecological point of view, such concentrations can already disrupt natural self‐purification processes and negatively affect biota [15]. According to the recommendations of the European Environment Agency, a TSS concentration of up to 10 mg/L is associated with good ecological status, while > 25 mg/L is characteristic of degraded water bodies [16]. Turbidity is a sensitive indicator of the overall impact of suspended solids, including colloidal particles and dissolved organic matter [17]. According to World Health Organization recommendations for drinking water, the turbidity standard should not exceed 5 nephelometric turbidity units (NTUs) and optimally should be < 1 NTU. In the context of ecosystem standards, values above 10–15 NTUs already indicate excessive load [18]. In addition, high turbidity complicates orientation and feeding for visual predators, affects fish behavior and migration, and can cause physical damage to the gills of aquatic organisms, particularly fish and mollusks [19]. Similarly, the transparency index, measured using a Secchi disk (or Snellen cylinder), is used to assess eutrophication: Values below 10–15 cm (Snellen) are often associated with high trophic status, massive phytoplankton growth, or anthropogenic pollution. The threshold values presented in this review should not be interpreted as universal ecological limits. Suspended‐solids and turbidity criteria are context‐dependent and may differ according to the intended water use, ecosystem type, hydrological regime, species sensitivity, and regulatory jurisdiction. For example, drinking‐water turbidity criteria are established for human health and treatment considerations, whereas ecological turbidity or suspended‐sediment thresholds are generally associated with the protection of aquatic habitats and biological communities. Similarly, particle‐size criteria reported for spawning substrates are applicable to particular fish habitats and should not be generalized to all aquatic ecosystems. Accordingly, the numerical values cited in this review are presented as examples of regulatory or ecological benchmarks rather than globally applicable standards.

Differences in sample preparation and operational definitions can produce substantially different SPM or TSS values even when samples originate from the same water body. For gravimetric determination, the choice of filter material and nominal pore size, filtration volume, drying temperature, drying duration, and handling of retained material can affect the measured particulate mass. Similarly, field filtration at a nominal 0.45 μm cutoff operationally separates “dissolved” and “particulate” fractions, but this boundary does not represent an absolute physicochemical separation because colloids and very fine particles may pass through the filter. Consequently, comparisons among studies should explicitly report filter characteristics, filtration conditions, sample storage, drying protocol, and the operational definition used for the particulate fraction. Standardized reporting of these parameters is essential for interstudy and interlaboratory comparability.

### 2.2. PSD

The size of SPM particles determines their mobility, sedimentation rate, bioavailability, and ability to penetrate biological tissues [20]. The predominance of the fine fraction of SPM causes prolonged suspension of particles in the water column, active transport by currents, and penetration into the lower horizons of water bodies. The granulometric composition of SPM is determined, in particular, by the laser diffraction method, which allows the calculation of particle sizes [21]. Small‐sized SPM has a high specific surface area, which increases its sorption capacity for dissolved pollutants such as HMs, phosphates, and OPs. The environmental consequences of the fine‐grained composition of SPM include not only the formation of stable suspensions, but also a decrease in the efficiency of filtration and sedimentation systems in natural and engineered environments. In addition, these particles are easily accessible for phagocytosis by filter feeders, which increases the risk of bioaccumulation of pollutants [22]. Environmental standards regulate the maximum increase in suspended‐particle concentration relative to background values, with a maximum increase of 5 mg/L over a weekly period for long‐term impacts, as well as the maximum short‐term increase in turbidity by 8 NTUs [23].

Ecological standards for SPM levels in aquatic ecosystems are based on an assessment of their impact on the physical and chemical conditions of the environment, in particular, gas exchange, bottom substrate porosity, and the vital activity of aquatic biota [24]. In spawning grounds of fish species sensitive to siltation (in particular salmonids), maximum permissible proportions of fine fractions have been established: The proportion of particles with a diameter of less than 2 mm should not exceed 10%, < 3 mm—19%, and < 6.35 mm—25% of the total volume of the bottom substrate. These limits prevent excessive compaction of the gravel bed and ensure effective diffusion of dissolved oxygen to the eggs and larvae. The presence of a large number of finely dispersed fractions (< 50 μm) with a high specific surface area and the ability to sorb toxicants increases the risk of bioaccumulation of pollutants and reduces the effectiveness of filtration barriers in natural and artificial ecosystems.

### 2.3. Chemical Composition

The chemical composition of SPM is an important determinant of its environmental behavior and ecological significance. Unlike a chemically uniform phase, SPM represents a heterogeneous assemblage of mineral particles, organic material, biogenic residues, microorganisms, and, in impacted systems, particle‐associated contaminants. The relative contribution of these components varies with hydrological conditions, season, catchment characteristics, biological productivity, sediment resuspension, and anthropogenic inputs [25–28]. Consequently, the chemical characterization of SPM should distinguish between the intrinsic particulate matrix and substances that are associated with particle surfaces or incorporated into the particulate material. From an analytical perspective, this distinction is particularly important because the presence of a contaminant in the particulate fraction does not necessarily indicate the same chemical form, mobility, or biological availability. Particle‐associated substances may be retained within mineral structures, bound to oxide surfaces, associated with organic matter, or adsorbed onto the external surfaces of particles. Changes in pH, redox conditions, ionic strength, dissolved organic matter, and hydrodynamic regime may alter these associations and promote redistribution between particulate and dissolved phases [29–37]. The chemical composition of SPM is therefore closely linked to both contaminant transport and the potential for subsequent release or bioaccumulation.

#### 2.3.1. Natural Mineral and Organic Matrix

The natural particulate matrix of aquatic SPM is composed predominantly of mineral and organic constituents. Mineral phases commonly include aluminosilicates, iron and manganese oxides, silica, and carbonates, whereas the organic fraction comprises humic substances, plant and animal residues, detritus, extracellular organic material, and microbial biomass. The relative abundance of these components depends strongly on watershed geology, soil erosion, sediment inputs, aquatic productivity, and seasonal hydrological conditions. Aluminosilicate minerals, including clay and clay‐associated materials, represent an important component of fine‐grained SPM. Their environmental significance is related not only to their abundance but also to their relatively high specific surface area and surface charge, which provide sites for the retention of dissolved substances. Iron and manganese oxides are likewise important reactive phases because their surfaces can interact with dissolved metals and other chemical species. Silica‐containing particles may originate from weathering products, suspended sediment, and biogenic sources, while carbonate minerals are associated with the geological characteristics of the catchment and the geochemical conditions of the water body [25].

The organic fraction of SPM is highly variable and may include both autochthonous and allochthonous material. It can originate from phytoplankton, macrophytes, terrestrial plant residues, soil organic matter, microbial biomass, and partially decomposed detritus. Reported organic matter contents in SPM may range from approximately 5% to 40%, depending on season, biological productivity, hydrological conditions, and anthropogenic pressure [26]. Such variability is important because organic matter can modify particle density, aggregation behavior, surface properties, and the partitioning of hydrophobic contaminants between particulate and dissolved phases. In productive aquatic systems, seasonal increases in particulate organic matter may accompany periods of intense primary production and algal development. Accordingly, elevated concentrations of organic particulate material and associated OPs have been reported during summer–autumn periods in systems affected by algal blooms [27]. By contrast, periods of increased erosion and sediment mobilization may favor the mineral fraction, particularly where bank erosion, bottom‐sediment disturbance, or catchment runoff represents an important source of suspended material. Microorganisms constitute another biologically active component of the SPM matrix. Bacteria, algae, fungi, and other microorganisms may occur as individual cells or as components of organic aggregates and biofilms. Their presence can modify particle surface properties and contribute to the transformation and cycling of organic and inorganic substances. Detrital particles and microbial aggregates may also act as transitional structures linking dissolved organic matter with larger particulate material. Thus, the natural SPM matrix should not be regarded simply as an inert mineral phase but as a dynamic combination of mineral and biologically derived material. Anthropogenic activities can substantially modify this natural composition. Domestic and industrial wastewater discharges, agricultural runoff, urban drainage, transport‐related emissions, and atmospheric deposition may introduce additional mineral, organic, and contaminant‐associated material into aquatic systems [28]. These inputs may change both the absolute concentration of SPM and its chemical and surface characteristics, thereby affecting its capacity to retain and transport pollutants.

#### 2.3.2. Particle‐Associated Inorganic Contaminants

Among the most important contaminants associated with aquatic SPM are potentially toxic trace elements, including Pb, Cu, Zn, Cd, Cr, and Ni, together with other elements whose occurrence depends on geological and anthropogenic sources [29]. Their association with suspended particles may result from adsorption onto mineral surfaces, interaction with iron and manganese oxides, complexation with organic matter, precipitation of poorly soluble phases, or incorporation into particulate mineral structures. The distribution of trace elements between the dissolved and particulate fractions is controlled by both particle characteristics and the surrounding hydrochemical conditions. Fine‐grained particles are particularly relevant because their high specific surface area provides numerous potential binding sites. Iron and manganese oxide coatings and organic‐rich surfaces can further increase the capacity of SPM to retain trace elements. As a result, the particulate fraction can act as an important temporary reservoir and transport phase for metals within rivers, lakes, reservoirs, and estuarine systems [30].

The environmental significance of particle‐associated metals depends not only on their total concentration but also on their chemical association and subsequent mobility. Once incorporated into or adsorbed onto SPM, metals may be transported downstream and ultimately deposited in bottom sediments. Sedimentation therefore represents a temporary removal pathway from the water column rather than necessarily a permanent loss from the aquatic system. Under changing environmental conditions, previously deposited material may be remobilized, allowing associated contaminants to return to the water column [35–37]. This behavior has implications for biological exposure. Contaminated SPM may interact with benthic organisms directly through contact with particles or sediments, through ingestion, or indirectly following the release of dissolved metal species. The ecological consequences may become particularly relevant during periods when aquatic organisms are undergoing sensitive biological processes, including reproduction, early development, migration, or other phases associated with increased physiological vulnerability [31]. Exposure pathways can include ingestion of contaminated particles, physical contact with contaminated substrates, and deposition of contaminated material in habitats used for reproduction or development [32]. These processes illustrate why measurements of bulk SPM concentration alone may be insufficient for evaluating environmental risk when the particulate fraction contains substantial amounts of potentially toxic elements.

#### 2.3.3. Particle‐Associated Organic Contaminants

SPM also provides an important carrier phase for particle‐associated organic contaminants, particularly compounds with relatively high hydrophobicity. The principal groups considered in aquatic environmental studies include polycyclic aromatic hydrocarbons (PAHs), polychlorinated biphenyls (PCBs), pesticides, phthalates, and other hydrophobic organic compounds [29]. These substances may enter aquatic environments through industrial and municipal discharges, agricultural runoff, atmospheric deposition, urban drainage, and the degradation of contaminated materials. The association of hydrophobic organic compounds with SPM is strongly influenced by the organic fraction of the particles and by the physicochemical properties of the individual compounds. Fine particles with a high specific surface area can provide extensive sorption surfaces, while particulate organic matter may contribute additional partitioning capacity for hydrophobic substances. Consequently, the concentration of an organic contaminant in the particulate phase may vary considerably according to particle size, organic carbon content, mineral composition, and the concentration and properties of the contaminant itself. Particle‐associated organic contaminants may subsequently be transported with suspended material and transferred to bottom sediments during periods of reduced water velocity. This creates a potential mechanism for their long‐term retention in sediment deposits and for later remobilization during high‐flow events, storms, tides, navigation, dredging, or other forms of sediment disturbance [36, 37]. Resuspension can therefore increase the environmental relevance of historically accumulated contamination by returning both particulate material and associated pollutants to the water column. The association with particles does not necessarily mean that a contaminant is permanently unavailable to organisms. Sorption may reduce the concentration of freely dissolved substances in the water column and thereby alter immediate exposure, but changes in pH, redox conditions, dissolved organic matter, ionic strength, or hydrodynamic conditions can modify particle–contaminant interactions and promote desorption or redistribution [33–35]. The resulting changes in contaminant partitioning can influence exposure pathways and contribute to secondary contamination of the aquatic environment. For this reason, assessment of organic contaminants in SPM should ideally distinguish between their total particulate concentration and their phase distribution. Analytical approaches that combine particulate separation with chemical determination can provide more informative evidence than measurements performed exclusively in the bulk water phase. Such an approach is particularly relevant for contaminants that have a strong tendency to associate with fine particles and organic‐rich particulate material. The accumulation of potentially toxic metals and OPs in particulate material also provides a rationale for considering sediment quality when evaluating the longer term consequences of SPM contamination. Sediment quality guidelines (SQGs) commonly use threshold‐based approaches to distinguish concentrations associated with a lower probability of adverse effects from those at which adverse biological effects become more likely. The threshold effect level (TEL) and probable effect level (PEL) are examples of such sediment‐quality benchmarks [38]. The values presented in Table 1 are therefore relevant for interpreting the potential significance of contaminants associated with particulate material that is subsequently deposited in bottom sediments. They should be regarded as sediment‐quality benchmarks rather than universal concentration limits for SPM in the water column.

**TABLE 1:** Standards for potentially toxic trace elements and particle‐associated organic contaminants concentrations in bottom sediments.

HM or OP	TEL (mg/kg)	PEL (mg/kg)
Pb	35	100
Zn	120	410
Cu	34	197
Cd	1	5
Cr	40	111
Ni	26	55
Surface‐active substances	—	> 600
Polychlorinated biphenyls	10	50

The occurrence of metals and OPs in SPM therefore has implications extending beyond the instantaneous quality of the water column. Suspended particles can facilitate downstream contaminant transport, contribute to sediment contamination after deposition, and subsequently become a source of secondary pollution following resuspension [36, 37]. Integrated assessment should consequently consider both particulate concentrations and the chemical characteristics of the material carrying the contaminants.

#### 2.3.4. MPs as Anthropogenic Particulate Matter

MPs represent a distinct anthropogenic particulate component that can occur within suspended material in both freshwater and marine environments. They are conventionally defined as plastic particles smaller than 5 mm and may enter aquatic systems as intentionally manufactured small particles or as secondary particles generated through the fragmentation of larger plastic materials [39, 40]. Their occurrence within SPM is environmentally significant because their physical and chemical characteristics differ substantially from those of naturally occurring mineral and organic particles. MPs can be introduced directly into aquatic environments as primary particles, including industrial plastic pellets and other manufactured particles, or generated secondarily through the weathering and fragmentation of larger plastic debris. Ultraviolet radiation, mechanical abrasion, environmental weathering, and biological processes contribute to the progressive breakdown of larger plastic materials into smaller fragments and fibers [41]. The resulting particles may differ considerably in polymer type, size, shape, surface condition, and density. The transport behavior of MPs is therefore strongly dependent on their physical properties and interactions with the surrounding particulate matrix. Low‐density polymers may remain in suspension or near the water surface for extended periods, whereas denser particles or particles that become incorporated into mineral or organic aggregates may undergo enhanced settling [42, 43]. Aggregation with natural SPM can consequently modify the transport and residence time of MPs and influence their distribution between the water column and bottom sediments.

From an analytical perspective, characterization of MPs within SPM requires consideration of several complementary properties. Polymer type determines many of the particle’s physicochemical characteristics and can be assessed using spectroscopic approaches such as FTIR or Raman spectroscopy. Particle size is important for transport, aggregation, and biological uptake, whereas morphology, including fragments, fibers, films, and other forms, provides information on particle origin and environmental transformation. These characteristics should ideally be reported together because size alone does not adequately describe the behavior of anthropogenic particulate material. MPs may also interact with other contaminants present in aquatic environments. Their surfaces can provide sites for the association of hydrophobic organic compounds and trace elements, although the extent and environmental importance of these interactions depend on polymer characteristics, surface aging, biofilm formation, contaminant properties, and surrounding water chemistry [44, 45]. Weathered plastic surfaces may acquire altered chemical and biological properties that differ from those of newly manufactured polymers. Consequently, MPs can function as additional particulate carriers within contaminated aquatic systems rather than representing an isolated pollution category. The widespread occurrence of polyethylene (PE) and polypropylene (PP) is partly related to their extensive use in consumer and packaging materials. Their relatively low density can favor persistence within the water column and facilitate transport through aquatic systems, although actual environmental distribution depends on particle size, aggregation, biofouling, and interactions with mineral and organic SPM [46]. The biological significance of MPs is also related to their potential ingestion by aquatic organisms. Ingestion and other forms of exposure may result in physical interactions with biological tissues and may be accompanied by cellular or physiological responses, although the magnitude and ecological relevance of these effects vary with polymer type, particle size, morphology, exposure conditions, and organism [47]. Accordingly, MPs should be considered within SPM characterization as a specific anthropogenic particulate fraction, rather than simply as another chemical contaminant. Their environmental assessment requires simultaneous consideration of polymer composition, particle size and morphology, abundance, spatial and temporal distribution, and associations with other contaminants. Incorporating these parameters into SPM monitoring would provide a more complete representation of anthropogenic particulate pollution and its potential contribution to contaminant transport and ecological exposure.

### 2.4. Interaction of Factors

SPMs that combine fine particle size and the presence of sorbed toxicants pose the highest environmental risk. In such cases, pollutants remain in the aquatic environment for longer, are actively transported, pass through filtration barriers, and accumulate in food chains [48]. These characteristics increase the threat to aquatic fauna and create conditions for long‐term, cumulative effects. Thus, in environmental monitoring and assessment of the state of water bodies, it is extremely important to consider not only the mass concentration of SPM, turbidity, or transparency indicators, but also the size‐phase structure and chemical characteristics of SPM, which allow accurate identification of pollution sources, model the pathways of pollutant spread, and develop targeted remediation strategies [49–52].

## 3. Gravimetric and Optical Methods of Quantitative Analysis

Gravimetric determination of SPM is based on weighing a preweighed filter before and after filtration, followed by drying the retained particles at 103°C–105°C to a constant weight (Figure 3a). It is recommended to use glass fiber or membrane filters, maintain a controlled drying temperature, and ensure weighing accuracy of ±0.1 mg or better. The sample volume (usually 100–1000 mL) is selected so that the resulting retained mass exceeds the detection limit of the analytical method. The quantitative indicator of SPM content in gravimetric determination is TSS. According to standards [53, 54], samples should be stored at 4°C and analyzed no later than 48 h to prevent biological changes. The advantages of the method are high reproducibility and direct physical interpretation of the results, but accuracy is affected by humidity, temperature, drying conditions, and mechanical damage to the filters. Study [55] shows that even minor errors during weighing or sample processing can lead to significant deviations in concentration determination.

**FIGURE 3:**
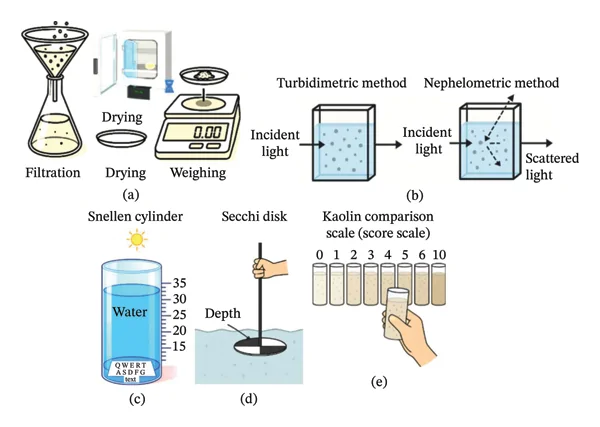
Gravimetric and optical methods of SPM.

Optical methods for assessing water transparency and turbidity are based on the interaction of light with suspended particles in the medium [56]. Unlike the gravimetric approach (direct mass assessment), optical methods measure the optical properties of a sample—the degree of light scattering and/or transmission and are therefore expressed in specialized units. In particular, the units of measurement are NTU [57], formazin nephelometric units (FNUs) [58], and transparency (%T, cm Snellen, m Secchi, or points) [59]. Conversion between different units of measurement is only possible if there is a calibration curve for a specific type of SPM. Depending on the nature of their implementation, they are conventionally divided into instrumental and visual observation methods. Turbidity is an optical property of a liquid that describes the degree of light scattering and absorption by particles in it. Turbidity is often assessed as a light scattering index and expressed in NTU, FNU, or light transmission in %T [60]. Depending on this, various methods can be used: nephelometric, which measures light scattering, and turbidimetric, which measures the intensity of light passing through a suspension formed by particles of the substance under study in a liquid (Figure 3b). Different devices may use different optical approaches, including photometric transmission, laser‐based, and infrared (IR) methods (Table 2). Transparency is an optical characteristic that shows the degree of light transmission and can be measured instrumentally using a photometer and expressed as %T, or visually using a Snellen cylinder (Figure 3c), a Secchi disc (Figure 3d), or Kaolin comparison scale balls (Figure 3е). The Snellen cylinder is a visual method for assessing water transparency. It is based on determining the height of the water column through which a standardized text or font remains visually readable. The result is expressed as the height of the water column, typically in centimeters. The method has been applied in pond monitoring, wastewater treatment, and water‐treatment studies [61–64]. Although less precise and more observer‐dependent than instrumental optical methods, it provides a simple and inexpensive approach for rapid assessment of water transparency. Kaolin comparison scales constitute one of the earliest approaches to turbidity assessment. The method relies on visual comparison of a water sample with reference suspensions containing known concentrations of kaolin particles. Despite limited precision and observer dependency, the approach remains useful for rapid field classification where instrumental measurements are unavailable. Calibration of optical instruments should account for the particle concentration range, PSD, composition, color, refractive index, and aggregation state of the target suspension. Measurement uncertainty may arise from instrument repeatability, calibration‐standard uncertainty, optical interference, sample heterogeneity, and temporal changes in particle characteristics. Therefore, calibration should ideally use representative local suspensions and be periodically verified against an independent reference measurement.

**TABLE 2:** Comparative characteristics of optical and visual methods used for water clarity and turbidity assessment.

Method	Principle	Units	Typical sensitivity	Disadvantages
Turbidimeter	Measures light interaction	NTU, FNU, %T	0.01–1000 NTU (high‐end 0.01 NTU)	Requires calibration (formazin), affected by color/refractive index, needs optical window maintenance
Nephelometer	Detects light scattered at 90° from incident beam	NTU or FNU	0.01–1000 NTU (high‐end 0.01 NTU)	Sensitive to particle type and color; needs calibration (formazin)
Laser/IR optical sensors	Use coherent (laser) or IR light and measure forward/backscatter or backscatter intensity; often for in‐line/remote sensing	NTU or FNU	< 0.01 NTU	Higher cost, complex maintenance
Photometric method (spectrophotometer/photometer)	Measures transmitted light or optical density (absorbance/optical density, OD) at a chosen wavelength; %T (absorbance conversion used to express transparency)	T, A, optical density; may be empirically converted to NTU or mg/L	Instrument‐dependent; precision often reaches 0.001	Strongly affected by sample color and dissolved substances
Secchi disk	Visual disappearance/appearance depth of a disk lowered in the water column	Meters	Meter‐scale resolution	Observer‐dependent, affected by sun/waves, not precise for low turbidity
Snellen cylinder	Visual test target at cylinder base; measure water column height at which target becomes just visible	Centimeters	Centimeter‐scale resolution	Subjective, depends on lighting and observer acuity
Kaolin comparison scale (score scale)	Visual comparison of sample against series of kaolin suspensions with increasing turbidity; assign a score (0–10)	Arbitrary score/points	Coarse; suitable for rough field classification	Subjective, low repeatability

Laser and IR optical sensors represent an advanced extension of nephelometric techniques. These instruments employ coherent laser beams or IR radiation and quantify forward‐scattered, side‐scattered, or backscattered light generated by suspended particles. Owing to their high sensitivity and rapid response, such sensors are widely applied in continuous monitoring systems, autonomous buoys, and remote environmental observation networks. Photometric methods are based on the attenuation of transmitted light through a water sample and are commonly expressed as absorbance (A), optical density (OD), or percent transmittance (%T). The approach follows the Beer–Lambert relationship and is particularly useful for laboratory investigations where relative changes in particle concentration are of interest.

The analytical techniques used for transparency and turbidity assessment differ substantially in terms of sensitivity, operational requirements, and applicability under field conditions (Table 2). Instrumental optical methods, including turbidimeters, nephelometers, laser‐based sensors, and photometric systems, provide quantitative measurements with high precision and are suitable for automated and continuous monitoring. Among these techniques, laser and IR sensors exhibit the highest sensitivity, whereas nephelometric measurements remain the most widely accepted approach for low‐turbidity waters. In contrast, visual methods such as the Secchi disk, Snellen cylinder, and kaolin comparison scale require minimal equipment and operational costs, making them attractive for rapid field surveys and long‐term observational programs. However, their performance is strongly influenced by environmental conditions and observer‐related variability. Consequently, the selection of a particular method depends on monitoring objectives, expected turbidity range, required analytical accuracy, and available technical resources.

Turbidimetric and nephelometric methods are optical quantitative analytical methods based on measuring the interaction of light with particles in a liquid. In the simplest version of turbidimetry, the intensity of transmitted light is evaluated; in the nephelometric version, the intensity of scattered light at an angle (usually 90°) is evaluated. The result is presented as a value reflecting turbidity (NTU or FNU) or as a percentage of transmitted light (%T), and usually correlates with the TSS concentration in the presence of a calibration curve [65]. A typical turbidimeter/nephelometer consists of a light source (lamp, white emitter, or laser), an optical cell or transparent sample container with a defined optical path, photodetector(s) (photoelectric detectors/photodiodes) located at a specified angle(s) relative to the incident beam, electronics for signal amplification, calibration, and interpretation [59]. Various sources are used as light sources in nephelometers and turbidimeters. In particular, white light/tungsten/xenon were used in classical devices [66, 67], which describe the configurations of devices that can use a polychromatic source and detectors for 90° scattering. Laser diodes provide high beam coherence and a good signal‐to‐noise ratio at low concentrations [68]. EPA Method 180.1 [68] and ISO 7027‐1 [69] provide standardized procedures for turbidity measurement and define two quantitative methods using optical turbidimeters or nephelometers to determine water turbidity. Instrumental data can also be integrated into buoys and autonomous platforms; turbidimeters are often combined with satellite observations for spatiotemporal turbidity maps [70]. Recent work describes open‐source and low‐cost LED dual‐detector sensors for mass deployment in river monitoring networks, enabling denser spatial monitoring. Turbidimetric and nephelometric methods are rapid and allow for continuous monitoring and automation. However, they have limitations, in particular, the influence of water color, as color can distort signals, especially for visible LEDs and white light sources. Furthermore, the size, shape, and aggregation of SPM affect the intensity of scattering.

Although visual observation methods are less accurate and more subjective than instrumental methods, they provide an immediate and rapid indication of environmental conditions that is useful for long‐term time series and large‐scale surveys, especially in conditions of limited resources or high observation point density. The Secchi disk (Secchi depth) is a historically established method for measuring the transparency of open water bodies, which involves lowering a marked disk into a water column and recording the depth at which the disk disappears from view and reappears (Figure 2d). Secchi depth integrates the total attenuation of light along the vertical and is widely used in marine and freshwater ecology as a simple indicator of trophic status, transparency for photosynthesis, and for long‐term observation series (in particular for assessing changes due to erosion, human activity, or climatic influences). Despite its practicality, Secchi results are sensitive to observation conditions (sun, cloud cover, waves, and operator technique), so publications recommend standardizing the procedure (side of the vessel, disc diameter, time of day) and documenting metadata for comparability [71].

The Snellen cylinder is a laboratory and field method in which a water sample is placed in a transparent graduated cylinder with a contrasting target (text or mark) at the bottom; transparency is determined as the height of the water column at which the test pattern becomes just visible (Figure 2c). This approach is suitable for semiquantitative assessment of individual samples in field or stationary laboratory conditions and allows work with small volumes and quick comparison of samples with each other. The method is susceptible to observer bias and lighting variations, so to improve reproducibility, it is recommended to use standardized targets, a constant source of diffused light, and replicate measurements. In technical descriptions and regional protocols, the Snellen cylinder is often recommended as an affordable tool for assessing natural waters and wastewater [61]. The Kaolin comparison scale is a visual comparison method based on a set of reference kaolin suspensions with a stepwise turbidity scale (e.g., 0–10 points); the operator compares the test sample with the references and assigns the closest level (Figure 2d). Due to its low cost and simplicity, this method has historically been used for rapid inventory and field observations where instruments are not available. However, it is highly dependent on the preparation of standards (kaolin settles and must be thoroughly mixed), lighting, and observer experience, and provides a rough gradation that can only be linked to NTU or mg/L after local calibration, if necessary. Today, kaolin‐based scales are sometimes used as a backup or preliminary technique in laboratories and serve as a teaching tool to facilitate the interpretation of optical data. The scientific literature provides numerous examples of the use of these visual methods in monitoring aquatic ecosystems: Secchi depth is systematically used in long‐term observation series to assess changes in the transparency of lakes and marine areas and for integration with biogeochemical indicators [72, 73]; the Snellen cylinder is used in laboratory and sanitary protocols for initial and intermediate sample testing [62–64]; and kaolin scales have been used in field surveys and as an auxiliary gradation for rapid assessment of changes in rivers and tributaries. There are also examples of combining traditional visual methods with modern inexpensive sensor platforms to expand the spatial and temporal density of observations [74, 75].

Optical measurements (NTU, FNU, %T, Secchi depth, Snellen, kaolin score) correlate with the TSS mass concentration, but there is no universal conversion formula. The relationship between turbidity and suspended‐solids concentration is frequently described by the generalized power function (1). Importantly, relationships between optical measurements and TSS are site‐ and matrix‐specific and should not be generalized beyond systems for which appropriate local calibration has been established:

(1) TSS=k·NTUn,

where *k* and *n* are calibration coefficients determined for a specific aquatic system. For systems exhibiting an approximately proportional response, the relationship may be simplified to a linear model.

The NTU/FNU relationship with TSS exists and is often strong at the level of a single site/region, but it is not universal; the model form and coefficients depend on the physicochemical properties of the suspension and the hydrological context. Many studies report good correlations between turbidity (NTU/FNU) and TSS within a given site or watershed (sometimes *R* ^2^ > 0.9 for log‐transformed fits), but the regression parameters vary widely between sites and hydrological conditions [76]. Among the main reasons for the impossibility of unifying the conversion of quantitative units of measurement of TSS are PSD, mineral vs organic composition (refractive index), organic matter/chlorophyll (color), instrument geometry/wavelength (NTU vs FNU), and flow/event conditions [77, 78]. T, Secchi depth, Snellen transparency, and kaolin scores may correlate with TSS, but such relationships often require multidimensional models. General relationships between quantitative characteristics of TSS content using the example of the Styr River water are shown in Figure 4 [79, 80]. The logarithmic dependence of TSS on NTU (Figure 4a) confirms that turbidity in nephelometric measurement is an adequate basis for expressing mass concentration within the studied range; similarly, FNU correlates with NTU almost proportionally, which indicates the consistency of measurements under different standards (Figure 4b). Light transmittance (%T) and Secchi depth (cm) show an exponential decay with increasing TSS (Figure 4c,d), which corresponds to the physical interpretation of dominant scattering in the system, but the nonlinearity of the curve indicates a decrease in sensitivity at very high concentrations and the possible influence of colored substances. Secchi depth (m) decreases with increasing TSS (Figure 3e), although its value also depends on the color of the solution. Secchi depth is a more representative indicator in a certain range because it has a longer linear section. Finally, the visual kaolin scale shows a correlation with TSS/NTU (Figure 4f), but the wide spreads in each score highlight the methodological uncertainty of this approach. Overall, the proposed graphs are mutually consistent, but any conversions between optical units and TSS should be made through local calibration models and with explicit consideration of uncertainty intervals [81].

**FIGURE 4:**
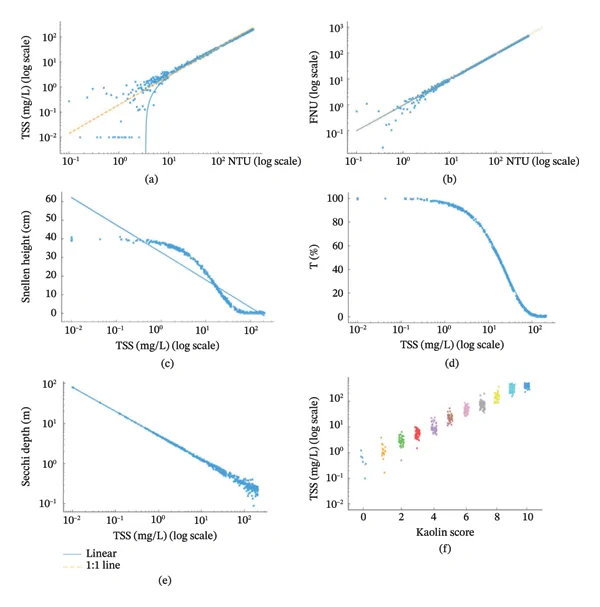
Relationships between TSS concentration and optical water‐quality parameters determined using Styr River water samples: (a) TSS versus turbidity (NTU), (b) comparison between NTU and FNU turbidity measurements, (c) settling height as a function of TSS concentration, (d) light transmittance (T) versus TSS, (e) Secchi depth versus TSS, and (f) calibration of TSS concentration using kaolin score classes.

Turbidity should be treated as a surrogate measurement of suspended particulate concentration rather than as a direct measurement of TSS. Calibration should therefore be performed using representative water samples covering the expected range of particle concentrations and hydrological conditions. Ideally, turbidity measurements should be paired with gravimetric TSS determinations collected over low‐flow, high‐flow, and event‐driven conditions. Matrix effects may arise from differences in particle mineralogy, organic matter content, color, particle shape, aggregation state, and refractive index. These effects can cause the same TSS concentration to generate different turbidity responses among sampling locations or seasons. Accordingly, turbidity can reliably substitute for gravimetric TSS only when a stable site‐specific or matrix‐specific calibration relationship has been demonstrated and periodically validated. A single universal NTU‐to‐mg L^−1^ conversion should not be assumed. Gravimetric and optical methods of quantitative SPM analysis complement each other in terms of measurement principle and practical application. The gravimetric approach, based on weighing the sample after filtration and drying, provides a direct mass estimate of TSS content with high reproducibility, but requires strict control of drying conditions, weighing, and filter preparation. Optical methods (turbidimetric, nephelometric, photometric, as well as visual observation methods—Secchi disk, Snellen cylinder, kaolin scale) are based on measuring light scattering and/or transmittance and report results in NTU, FNU, %T, or conventional points. They provide fast, automated, and continuous determination of transparency and turbidity, but are sensitive to the color, size, and shape of particles. Visual methods are less accurate, but remain useful for long‐term monitoring and field surveys with limited resources. There are often strong local correlations between optical parameters and TSS, but there are no universal relationships due to the influence of the physicochemical properties of the suspension, instrument characteristics, and hydrological conditions. Therefore, conversion between measurement units requires a local calibration model that takes uncertainty into account.

## 4. Granulometric Analysis: Sedimentation, Settling, and PSD

Particles present in natural and engineered aquatic systems span a wide size spectrum, ranging from nanometer‐scale solutes to visible suspended solids. Their classification is typically based on size, physicochemical properties, and behavior during filtration or sedimentation. Particles larger than ∼25 μm are visible to the naked eye, while fractions below this threshold require microscopic or instrumental methods for detection (Figure 5a). SPM is operationally defined as the fraction retained by a 0.45‐μm membrane filter (≥ 0.45 μm). This category includes coarse dust, fibers, and pollen (∼10 μm), medium dust, plant spores, and mold (5–10 μm), as well as fine dust, bacteria, and dandruff (1–5 μm). Within the suspended fraction, particles can further be divided into settleable solids, which sediment under quiescent conditions and within 1 h in an Imhoff cone, and nonsettleable suspended solids, which remain in suspension. Colloids occupy the intermediate domain between dissolved and particulate matter and typically range from the nanometer to approximately micrometer scale. They may consist of organic macromolecules, humic substances, and inorganic colloids such as clays and metal oxides. Due to their small size and high surface reactivity, colloids play a critical role in trace metal transport and pollutant binding. Dissolved substances are conventionally defined as the fraction passing through a 0.45‐μm membrane (< 0.45 μm). This group includes truly dissolved solutes and colloidal‐dissolved species. While operational in nature, this classification remains the standard in water‐quality assessment and environmental monitoring, enabling consistent comparison across studies. The characterization of SPMs in natural waters requires detailed knowledge of PSD and sedimentation behavior. These parameters govern the transport of contaminants, the stability of suspensions, and the efficiency of water‐treatment processes [82, 83]. In many water‐quality protocols, the particulate fraction is operationally defined as material retained by a 0.45‐μm filter. However, this cutoff is not a universal physical boundary between particulate, colloidal, and dissolved phases. Alternative fractionation approaches include sequential membrane filtration, ultrafiltration, tangential‐flow filtration, centrifugation, sedimentation, and field‐flow fractionation, depending on the target particle‐size domain and analytical objective.

**FIGURE 5:**
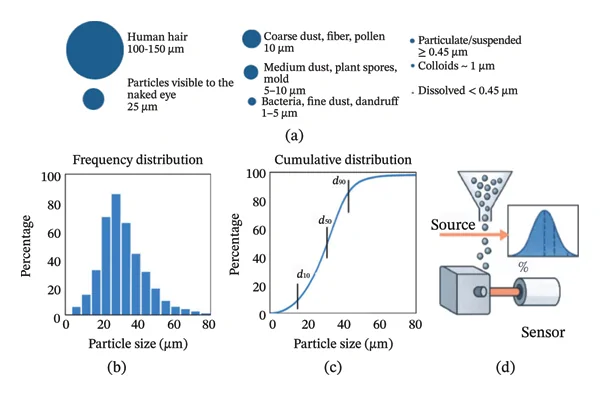
Classification of particles (a), characteristics of particle distribution measurements (b), (c), and the principle of the optical method (d).

Granulometric composition is typically described using PSD curves that reflect the relative frequency or cumulative percentage of particles across size intervals. Two major representations are employed [84]. Frequency distribution (Figure 5b) is expressed as histograms, showing the proportion of particles within defined size classes. The modal size (mode) indicates the most abundant fraction, while the distribution shape (symmetrical or skewed) provides insights into aggregation or fragmentation processes. The cumulative distribution (Figure 5c) is used to determine characteristic diameters such as *d*_10_ (10% finer), *d*_50_ (median diameter), and *d*_90_ (90% finer). These parameters are widely applied in hydrology, sedimentology, and environmental studies to classify suspended matter with respect to its mobility, settling potential, and sorption capacity [85]. The classification of sediment particles according to size and settling velocity provides essential insight into their transport and deposition behavior in aquatic systems. Coarse fractions, including boulders, cobbles, and gravels, are characterized by dimensions exceeding several millimeters and settle almost instantaneously in water. These materials are typically deposited close to their source and rarely occur in suspension under natural flow conditions. Sand particles, ranging from 2 mm down to approximately 62 μm, exhibit comparatively high settling velocities and therefore tend to accumulate rapidly, forming bed deposits within short transport distances. In contrast, finer sediments such as silts, with sizes between 62 and 4 μm, demonstrate markedly lower rates of sedimentation [86]. These particles may remain in suspension for extended periods, especially under turbulent flow regimes, which promotes their downstream transport. The finest fraction is represented by clay minerals, with particle diameters smaller than 4 μm. Due to their extremely slow settling velocities and the influence of colloidal interactions, clays can persist in suspension almost indefinitely (Table 3). This property makes them a dominant carrier phase for trace metals, organic contaminants, and other pollutants in natural waters [87, 88]. Validation of optical particle‐sizing results should involve comparison with an independent reference technique selected according to the particle‐size range. Laser diffraction measurements may be cross‐checked using microscopy or image analysis for larger particles, while submicrometer measurements may require complementary techniques such as DLS, nanoparticle tracking analysis, or electron microscopy. Replicate measurements, recovery tests where applicable, and assessment of intermethod agreement are important for identifying systematic biases caused by particle shape, aggregation, refractive‐index assumptions, and polydispersity.

**TABLE 3:** Generalized particle‐size classes and their theoretical settling velocities.

Particle group	Size (mm) (μm)	Settling velocity (s/cm)
Boulders	> 256	—
Cobbles	256–64	—
Gravel (coarse–fine)	64–2	—
Sand (very coarse–fine)	(2000‐62)	< 1–1000
Silt (coarse–very fine)	(62‐4)	3–700
Clay (coarse–very fine)	(< 4)	700–11.000

The settling behavior of particles is generally described by Stokes’ law, which links settling velocity to particle diameter, density, viscosity of the medium, and gravitational acceleration [89]. Under laminar flow conditions, which are generally applicable to particles up to approximately 100 μm, the sedimentation velocity increases quadratically with diameter, meaning that doubling the particle size reduces the settling time by approximately a factor of four. For colloidal fractions (< 1 μm), intermolecular forces dominate, making natural sedimentation inefficient unless aggregation or flocculation occurs. In practice, deviations from Stokes’ predictions are common due to the heterogeneity of natural waters. Organic coatings, ionic strength, and dissolved substances may stabilize fine fractions or promote aggregation, altering effective settling velocities. Larger particles (> 63 μm) generally settle more rapidly than fine ones (< 8 μm), but during sedimentation, the PSD of resuspended material shifts dynamically, reflecting processes of aggregation, disaggregation, and selective deposition. Empirical studies confirm that particle settling behavior varies considerably with water type and particle characteristics. In seawater, loess particles exhibit rapid settling of coarse fractions, accompanied by PSD transformation from normal to skewed curves over time. Nanoparticles and natural colloids often undergo heteroaggregation, strongly affecting sedimentation rates [90]. Reported settling velocities for engineered nanomaterials in natural waters range from 0.0001 to 0.14 m/day, while heteroaggregation rates span from 0.007 to 0.6 L·mg^−1^ day^−1^, with the highest values observed in marine environments.

Optical methods can also provide fast, repeatable, and often real‐time particle‐size assessment (Figure 5c). Most optical diffraction and light scattering models are used to determine the premise that particles are spherical, homogeneous, and have a well‐defined refractive index. In natural waters, suspended solids often include irregular mineral fragments, organic detritus, flocs, and biological structures, which deviate significantly from spherical geometry. Such irregularities alter scattering patterns and may cause systematic errors in calculated particle diameters. Additionally, variations in refractive index among heterogeneous particle mixtures complicate the application of optical models. Organic coatings, adsorbed ions, and internal porosity modify light interaction, resulting in under‐ or overestimation of particle size. Heterodisperse suspensions, such as those containing both clay minerals and MPs, present further challenges, since scattering signals overlap across size ranges.

Laser diffraction is one of the most widely used techniques for particle‐size analysis. When a coherent light beam passes through a suspension, larger particles scatter light mainly at small angles, whereas finer particles scatter at wider angles [91]. By interpreting these scattering patterns with diffraction models, PSDs can be reconstructed across a wide range, from nanometers to millimeters, with good precision and reproducibility. DLS complements this approach by targeting colloidal and submicron fractions. It measures fluctuations in scattered light intensity caused by Brownian motion, and from autocorrelation functions derives the hydrodynamic diameter of particles [92]. DLS is particularly effective for particles smaller than 1 μm, where sedimentation methods cannot be applied. IR and near‐infrared (NIR) sensors are increasingly used in environmental monitoring because of their robustness and suitability for in situ applications [93]. They rely on differences in absorption and scattering of IR light, allowing distinctions between suspended solids, colloids, and organic matter. Turbidimetric and nephelometric instruments, traditionally employed to estimate concentrations, can also yield indirect information on particle size. In particular, multiangle nephelometry uses the angular distribution of scattered light to distinguish size classes [94]. These optical devices are portable, energy‐efficient, and practical for field use in rivers, lakes, and treatment facilities. Despite these advantages, optical methods require careful calibration and often need validation against complementary techniques such as sedimentation tests, electron microscopy, or image‐based analysis to ensure reliable results.

PSDs obtained from different analytical techniques should not be assumed to be directly interchangeable. Laser diffraction derives particle size from angular light scattering patterns and commonly reports an equivalent spherical diameter. Sedimentation methods, in contrast, estimate size from settling behavior and therefore depend on particle density, shape, viscosity, aggregation, and hydrodynamic conditions. Microscopy provides direct information on particle morphology and projected dimensions but is sensitive to particle orientation, image resolution, segmentation criteria, and the number of particles analyzed. Consequently, the same natural‐water suspension may yield different apparent size distributions when measured by laser diffraction, sedimentation, and microscopy. These differences are particularly important for heterogeneous samples containing clay minerals, organic aggregates, biological particles, and MPs. Cross‐method comparisons should therefore report the measurement principle, size definition, dispersion protocol, and assumptions used for data processing.

## 5. Chemical Composition

The determination of the chemical composition of SPM in natural waters requires careful consideration of both separation procedures and analytical techniques. The initial step typically involves the isolation of particulate fractions from bulk water samples, which can be achieved by filtration through membrane filters of defined pore size, centrifugation, or sedimentation [95]. Filtration is the most widely applied approach due to its simplicity and reproducibility, although it may introduce artifacts related to filter adsorption or particle loss. Centrifugation provides an alternative means of concentrating SPM, particularly for fine and colloidal particles. Following isolation, the particulate phase generally requires decomposition prior to elemental analysis. Acid digestion using mixtures of nitric, hydrochloric, hydrofluoric, or perchloric acids under controlled heating is the most established method, ensuring efficient breakdown of mineral and organic matrices. Microwave‐assisted digestion has become increasingly popular because it offers shorter processing times, improved reproducibility, and reduced reagent consumption compared to conventional open‐vessel digestion. Ultrasonic‐assisted extraction has also been employed to enhance the release of loosely bound elements, although its efficiency for refractory minerals is limited. Each approach presents advantages and limitations: Conventional acid digestion provides complete decomposition but may involve high reagent use and contamination risks, whereas microwave‐assisted methods improve efficiency and safety, and ultrasonic treatment is best suited for preliminary leaching studies rather than total digestion.

In contrast to destructive techniques, certain analytical methods permit the direct characterization of SPM with minimal or no sample dissolution. X‐ray fluorescence (XRF) and energy‐dispersive spectroscopy (EDS) coupled with scanning electron microscopy allow qualitative and semiquantitative determination of elemental composition directly on solid particles, preserving information on particle morphology and surface distribution [96]. Similarly, X‐ray diffraction (XRD) provides insights into crystalline phases without chemical digestion. However, these methods are generally less sensitive for trace element determination compared to solution‐based techniques (Table 4). For the quantification of HMs, inductively coupled plasma mass spectrometry (ICP‐MS) and inductively coupled plasma optical emission spectrometry (ICP‐OES) remain the most sensitive and widely used methods after complete acid digestion [97]. ICP‐MS is among the most sensitive techniques currently available for determining trace elements associated with SPM. The method involves ionization of sample constituents within a high‐temperature argon plasma followed by mass‐to‐charge separation and detection of individual ions. Detection limits commonly reach the ng L^−1^ range, allowing accurate quantification of trace metals and metalloids even at extremely low concentrations. Graphite furnace atomic absorption spectrometry (GF‐AAS) also provides high sensitivity for selected metals, although throughput is lower. In the case of organic compounds associated with SPM, extraction with organic solvents followed by analysis using gas chromatography–mass spectrometry (GC–MS) or liquid chromatography–mass spectrometry (LC–MS) is commonly applied [98, 99]. Recent advances include pyrolysis GC–MS and thermal desorption techniques, which allow analysis of particulate‐bound organic matter with minimal preparation. Dynamic adsorption, desorption, and resuspension processes require analytical approaches capable of resolving both the particulate phase and changes in pollutant speciation or concentration. Sequential filtration combined with ICP‐MS or ICP‐OES can be used to track changes in dissolved and particulate metal fractions during controlled perturbation experiments. For organic contaminants, particle extraction followed by GC–MS or LC–MS provides information on changes in particle‐associated concentrations. XRD and XRF can help identify changes in mineralogical and elemental composition, whereas SEM–EDS provides spatial information on particle morphology and elemental associations. Particle‐size analysis by laser diffraction or microscopy is particularly useful when resuspension experiments are accompanied by aggregation, fragmentation, or selective mobilization of specific particle fractions. Combining these methods provides a more direct link between observed changes in SPM concentration and the mechanisms controlling contaminant mobilization.

**TABLE 4:** Comparison of methods for measuring chemical composition of SPM.

Goal	Sample preparation	Method	Advantages	Limitations/notes
Total metals in SPM	Digest filter or sediment	U.S. EPA Method 3050B (https://www.epa.gov/sites/default/files/2015-06/documents/epa-3050b.pdf), U.S. EPA Method 3051A (https://www.epa.gov/sites/default/files/2015-12/documents/3051a.pdf)	Robust multielement analysis; widely harmonized protocols	U.S. EPA Method 3050B does not completely digest silicate minerals; U.S. EPA Method 3052 requires hydrofluoric acid
Dissolved vs. particulate partitioning	Field filtration at 0.45 μm; acidification of filtrates	American Public Health Association Standard Method 2540D (https://www.testinglab.com/apha-2540d-total-suspended-solids-tss-test-in-groundwater), Standard Method 3030 (https://www.nemi.gov/methods/method_summary/4691/), U.S. EPA Method 3005A (https://www.epa.gov/sites/default/files/2015-12/documents/3005a.pdf)	Provides clear operational definitions consistent with regulatory frameworks	Filtration at 0.45 μm cutoff is an operational definition and does not perfectly separate dissolved vs particulate fractions
Colloids/nanoparticles (metals)	Asymmetric flow field‐flow fractionation or tangential flow filtration/cross‐flow filtration; minimal digestion	Asymmetric flow field‐flow fractionation coupled with ICP‐MS; single‐particle inductively coupled plasma mass spectrometry	Enables size‐resolved analysis, particle counting, and size distribution	Requires specialized instrumentation
On‐filter elemental mapping	None (analysis directly on dried filter)	XRF, scanning electron microscopy with energy‐dispersive X‐ray spectroscopy, laser ablation inductively coupled plasma mass spectrometry	Nondestructive options available; allows spatially resolved information	Susceptible to matrix effects; quantitative analysis requires appropriate standards
Polycyclic aromatic hydrocarbons and semivolatile organic compounds	Accelerated solvent extraction, Soxhlet extraction, or ultrasonic extraction	GC–MS according to U.S. EPA Method 8270D (https://19january2017snapshot.epa.gov/sites/production/files/2015-12/documents/8270d.pdf)	Well‐established and validated methods; large compound libraries available	Solvent and time‐intensive procedures
Particle‐bound polycyclic aromatic hydrocarbon	None	Thermal desorption gas chromatography–mass spectrometry	Faster analysis; requires less solvent	Potential bias due to volatility losses; requires careful calibration
Microplastics (identification and mass quantification)	None or mild cleaning of filters/samples	Fourier‐transform infrared spectroscopy, Raman microspectroscopy, pyrolysis–gas chromatography–mass spectrometry	Polymer‐specific identification; can target particles or bulk polymer mass	Have lower size limits for detection; pyrolysis GC–MS destroys particle morphology

SPM not only impairs aquatic ecosystem functioning but also complicates water management strategies. Therefore, the development of ecological regulation measures aimed at mitigating the negative effects of SPM has gained attention in recent years. Analytical monitoring provides the evidence base for evaluating the effectiveness of SPM mitigation and catchment‐management measures. For example, study [100] demonstrated that water regulation in drained lands can be optimized to enhance ecological efficiency under climate variability, thereby reducing the mobilization of suspended material into adjacent watercourses. Similarly, technical approaches such as drainage‐screened modules have been tested as effective tools for limiting horizontal water filtration, contributing to a reduction of fine particles entering water bodies [101]. These practices highlight that mitigation of SPM requires a combination of engineering interventions and ecological strategies that balance land‐use needs with environmental protection goals. Importantly, integrating such measures into catchment‐level management frameworks can significantly reduce the long‐term accumulation of SPMs and improve the resilience of aquatic systems to climatic and anthropogenic stressors. Long‐term assessments of drinking water reservoirs illustrate how sustained monitoring of trophic status trends can guide mitigation strategies, particularly under conditions of progressive eutrophication [102]. At the same time, behavioral dynamics of water‐quality parameters linked to trace metal exposure in lake ecosystems highlight the need for integrated approaches that account for both ecological processes and human health risks [103]. Eco‐phytoremediation techniques, such as the use of *Azolla microphylla*, demonstrate the potential of biological systems to enhance HM removal, improve water quality, and provide biomass cobenefits [104]. From a chemical perspective, mechanistic insights into adsorption processes, such as those involving hydroxyapatite, expand the understanding of dynamic interactions between SPM and pollutants, providing a basis for designing efficient remediation systems [105]. Groundwater vulnerability assessments further stress that regulatory frameworks should incorporate hydrogeological sensitivity alongside chemical monitoring, especially in rural areas where drinking water supply depends on shallow wells [106]. Complementary evidence from catchment‐scale studies confirms that both anthropogenic and natural pressures, including industrial zones and waste management facilities, exacerbate SPM impacts on water quality and demand adaptive management responses [107].

Optical turbidity measurements can provide high‐frequency observations for identifying temporal changes in SPM, whereas gravimetric TSS measurements can be used for calibration and validation. Geographic information systems (GIS) and remote sensing then extend these observations spatially, allowing analytical measurements to be linked to land use, hydrological conditions, and potential sediment sources. Recent advances in landscape and limnological research demonstrate the importance of integrating these approaches into SPM monitoring programs. GIS‐based approaches enable the spatial visualization of SPM dynamics, allowing researchers to track changes in transparency and turbidity across different hydrological conditions and land‐use settings. For instance, studies in the Cheremske Nature Reserve illustrated how satellite and field data can be combined for reservoir monitoring and ecological assessment [108]. Similarly, analysis of river runoff in the Sluch basin emphasized the role of extraterritorial impacts of hydraulic infrastructure in altering suspended‐sediment transport, which was effectively quantified through GIS‐supported modeling [109]. The application of cadastral and landscape modeling approaches has also been proposed as a prerequisite for sustainable recreational and conservation management of water bodies, highlighting the utility of GIS in linking geomorphology with aquatic ecosystem processes [110]. Furthermore, long‐term assessments of the Western Bug basin revealed that GIS technologies are particularly effective for capturing spatiotemporal changes in turbidity and other water‐quality indicators under combined anthropogenic and natural pressures [111]. Collectively, these studies underscore that GIS‐based monitoring is not only instrumental for mapping SPM dynamics but also provides a valuable framework for predictive modeling and environmental decision‐making. Recent developments in wetland mapping using advanced feature selection criteria with GF‐3 and Sentinel‐2 imagery highlight the growing potential of remote sensing for ecological applications, including the detection of water‐quality dynamics [112]. Beyond habitat classification, GIS‐based methods combined with water‐quality indices have also been applied to evaluate pollution sources and assess suitability for drinking purposes, often supported by machine learning tools that enhance predictive accuracy [113]. Similarly, groundwater studies demonstrate the capacity of GIS to integrate chemical analyses with spatial health risk assessment, particularly in regions where HM contamination poses a direct threat to public health [114].

The analytical techniques applied to SPM investigations differ substantially with respect to the particle‐size fractions that can be characterized, the types of analytes that can be determined, and the information generated regarding particle properties and contaminant associations. While gravimetric and optical methods are primarily used for quantification of suspended solids, particle‐sizing techniques such as laser diffraction and dynamic light scattering provide information on PSDs across different size fractions. Elemental characterization is commonly performed using ICP‐MS, ICP‐OES, XRF, or SEM–EDS, whereas FTIR, Raman spectroscopy, and chromatographic techniques are particularly valuable for molecular or polymer identification, including the characterization of organic contaminants and MPs [115]. XRD further complements these approaches by enabling mineralogical characterization of crystalline phases associated with suspended particles. The principal analytical capabilities, target analytes, and methodological limitations of the most widely applied techniques are summarized in Table 5. The practical requirements and applicability of these methods are summarized in Table 6.

**TABLE 5:** Particle‐size coverage, target analytes, and methodological limitations of analytical techniques applied to SPM.

Method	Effective particle‐size range	Target analytes/pollutants	Main advantages	Main limitations
Gravimetry	> 0.45–1 μm (filter‐dependent)	TSS	Standardized reference method; simple and inexpensive	Provides no information on particle composition, size, or contaminant content
Turbidimetry/nephelometry	∼0.1–1000 μm	Indirect estimate of suspended particles	Rapid, in situ, suitable for continuous monitoring	Strongly influenced by particle size, shape, color, and refractive index
Laser diffraction	∼0.02–2000 μm	PSD	Wide size range; rapid analysis; high reproducibility	Assumes equivalent spherical particles; no chemical information
DLS	∼1 nm–10 μm	Colloids, nanoparticles, nanoplastics	Highly sensitive to nanoscale particles	Poor performance in heterogeneous or polydisperse suspensions
ICP‐MS	Not size‐specific (after digestion)	Trace metals, metalloids, rare earth elements, toxic elements	Ultra‐trace detection capability; multielement analysis	Destructive; requires digestion; no particle‐size information
XRF	Typically > 1 μm particles	Major and trace elements	Minimal sample preparation; nondestructive	Lower sensitivity for light elements; matrix effects
XRD	> 1 μm crystalline particles	Mineral phases and crystalline compounds	Direct mineralogical identification	Ineffective for amorphous materials and low‐abundance phases

**TABLE 6:** Comparative characteristics of analytical methods used for SPM analysis in aquatic systems.

Method	Typical limit of detection	Cost	Analysis time	Automation potential	Field applicability
Gravimetry	∼0.1–1 mg L^−1^	Low	2–24 h	Low	High
Turbidimetry/nephelometry	∼0.01–0.1 NTU	Low	Seconds–minutes	High	Very high
Laser diffraction	∼0.02–2000 μm	High	5–15 min	High	Moderate
DLS	∼1 nm particles	High	5–20 min	High	Low
ICP‐MS	ng L^−1^–μg L^−1^	Very high	5–10 min/sample	Very high	Low
XRF	μg g^−1^	High	5–30 min	High	Moderate
Raman spectroscopy	μg–mg particle level	Very high	10–60 min	Moderate	Low

The analytical techniques applied to SPM investigations exhibit substantial differences in terms of sensitivity, operational complexity, analysis time, and applicability for environmental monitoring (Table 3). Gravimetric methods remain the standard approach for direct quantification of suspended solids because of their simplicity, low cost, and broad field applicability. Optical techniques, including turbidimetry and nephelometry, provide rapid measurements and are readily integrated into automated monitoring systems, making them particularly suitable for continuous water‐quality assessment. Particle‐size characterization methods such as laser diffraction and dynamic light scattering offer detailed information on PSDs over a wide range of particle dimensions, although their performance depends on sample heterogeneity and particle concentration. Advanced instrumental techniques, including ICP‐MS and XRF, provide highly detailed information on elemental and molecular composition but generally require specialized equipment, greater analytical expertise, and higher operational costs [116]. Consequently, the selection of an appropriate analytical approach depends on the objectives of the investigation, required analytical resolution, available resources, and the specific properties of SPM being examined.

Although the analytical techniques applied to SPM differ substantially in their measurement principles and target parameters, practical implementation is often determined by operational considerations, including analytical sensitivity, cost, processing time, degree of automation, and suitability for field deployment. As summarized in Table 6, gravimetric methods remain the most accessible and cost‐effective approach for routine quantification of suspended solids, whereas optical techniques provide rapid measurements and are readily integrated into automated monitoring systems. Advanced particle‐size characterization methods, including laser diffraction and dynamic light scattering, offer high analytical performance but require specialized instrumentation and controlled laboratory conditions. Techniques used for elemental and molecular characterization, such as ICP‐MS, XRF, and Raman spectroscopy, provide highly detailed compositional information, their broader application is often constrained by equipment costs, sample preparation requirements, and limited field applicability. Consequently, the selection of an analytical method represents a balance between analytical resolution, operational complexity, monitoring objectives, and available resources. Analytical performance is strongly method‐ and matrix‐dependent. Gravimetric methods provide a direct mass‐based measurement and remain suitable as reference procedures, but they are relatively labor‐intensive and do not provide information on particle size or composition. Optical methods offer substantially higher temporal resolution and are particularly advantageous for continuous monitoring. However, their response depends on particle size, shape, refractive index, color, and aggregation state. Laser diffraction provides broad particle‐size coverage and good reproducibility but relies on assumptions concerning particle geometry and optical properties. Chemical techniques such as ICP‐MS provide high sensitivity for trace elements but require sample digestion and do not preserve particle‐size information. Consequently, no single analytical technique provides a complete characterization of SPM, and method selection should be based on the analytical objective, particle characteristics, expected concentration range, and required temporal and chemical resolution.

## 6. Integrated Conceptual Framework and Multidimensional Risk Assessment of SPM

SPM represents a highly heterogeneous environmental matrix composed of mineral particles, organic matter, microorganisms, and anthropogenic contaminants that differ substantially in size, composition, origin, and environmental behavior. Consequently, no single analytical technique is capable of providing a complete characterization of SPM properties and associated environmental risks. The selection of an analytical approach depends largely on the specific objectives of the investigation, whether focused on concentration assessment, particle‐size characterization, elemental composition, or the identification of organic matter and MP contaminants. As illustrated in Figure 6, analytical methods applied to SPM studies can be organized according to four principal research objectives. Gravimetric and optical techniques are primarily used for the quantification of suspended solids and routine monitoring of water quality. Particle‐size characterization methods, including laser diffraction and dynamic light scattering, provide information on PSD and colloidal fractions that influence sedimentation processes, contaminant transport, and bioavailability. Chemical characterization techniques such as ICP‐MS and XRF enable the determination of elemental composition and the identification of contaminant sources. The complementary nature of these analytical approaches highlights the importance of integrated monitoring strategies. While concentration measurements provide information on the magnitude of particulate loading, particle‐size analysis describes the physical properties controlling transport and settling dynamics. Chemical and molecular characterization further reveal contaminant associations, pollutant partitioning, and potential ecological effects. Therefore, comprehensive assessment of SPM requires the integration of quantitative, physical, chemical, and molecular datasets within a unified analytical framework. Such multidimensional characterization is particularly important for environmental risk assessment because the ecological impacts of SPM are governed not only by its concentration but also by particle size, surface properties, contaminant load, and interactions with aquatic biota. The combined application of complementary analytical methods therefore provides a more robust basis for evaluating contaminant transport pathways, ecological exposure, and ecosystem responses than any individual technique alone.

**FIGURE 6:**
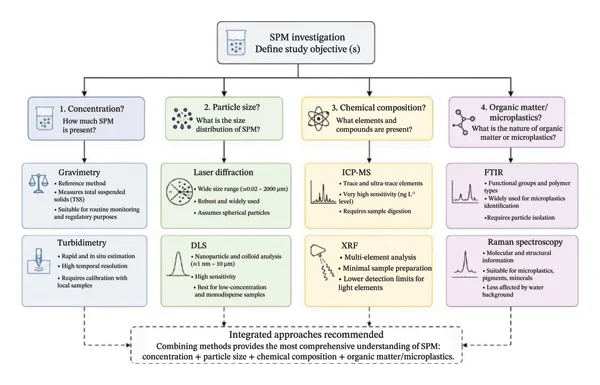
Decision framework for selecting analytical methods according to study objectives.

The integrated conceptual framework presented in Figure 7 synthesizes the multiple roles of SPM in aquatic systems by linking sources, physicochemical properties, transformation processes, and environmental consequences into a unified scheme. This model demonstrates that SPM should not be regarded solely as a passive indicator of water quality, but rather as an active mediator that controls the mobility, transformation, and bioavailability of contaminants. Natural and anthropogenic sources generate particles with distinct characteristics, which are subsequently modified by processes such as sorption–desorption, aggregation, sedimentation, and resuspension. These transformations directly influence pollutant transport, exposure pathways, and ecological effects, including bioaccumulation and secondary contamination. By integrating these components, the model provides a mechanistic basis for understanding how variations in SPM properties propagate through aquatic systems and ultimately determine ecological risk. Importantly, the conceptual model highlights the interdependence between PSD, chemical composition, and concentration, emphasizing that the environmental significance of SPM cannot be adequately assessed using a single parameter. Fine particles with high specific surface area and enriched contaminant loads represent a particularly critical fraction, as they remain suspended for extended periods and facilitate long‐range transport of hazardous substances. At the same time, larger particles contribute to sediment formation and act as reservoirs of pollutants that may be remobilized under changing hydrodynamic or physicochemical conditions. The conceptual framework presented in Figure 7 is a synthesis proposed by the authors based on the evidence identified in the scoping review. It is not a statistically validated predictive model and was not derived from a single experimental dataset. Rather, it integrates recurring relationships identified across studies concerning SPM concentration, PSD, chemical composition, transformation processes, and ecological consequences.

**FIGURE 7:**
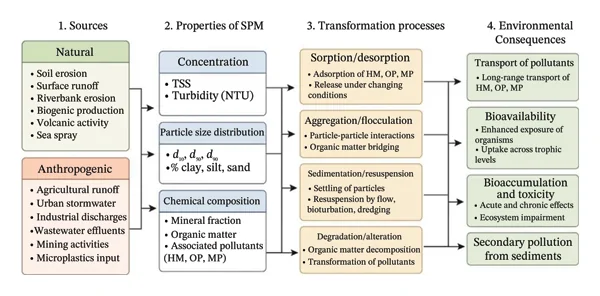
Integrated сonceptual model of SPM as a mediator of ecological risk.

Complementing this framework, the proposed multidimensional risk space provides a quantitative interpretation of SPM‐related risk by combining three key dimensions: concentration TSS, particle size (expressed as characteristic diameters such as *d*_50_), and chemical contamination (e.g., HMs, OPs, and MPs). This multidimensional representation allows the identification of distinct risk domains, including low‐risk conditions characterized by coarse, weakly contaminated particles, transport‐dominated regimes associated with fine particles of moderate concentration, and high‐risk zones defined by the coexistence of fine particle size and elevated contaminant loads. Such visualization reflects the nonlinear and synergistic nature of SPM impacts, where ecological risk emerges from the combined effect of physical and chemical properties rather than from any single variable. The proposed multidimensional space further provides a practical framework for interpreting monitoring data and supports the development of more robust environmental assessment strategies. By positioning observed conditions within this space, it becomes possible to distinguish between systems dominated by sedimentation processes and those characterized by persistent suspension and contaminant transport. This approach also facilitates the comparison of different water bodies and hydrological conditions, offering a standardized yet flexible tool for evaluating SPM dynamics across spatial and temporal scales. Together, the conceptual model and the multidimensional risk space form a complementary basis for advancing the understanding of SPM behavior and for improving the integration of analytical, ecological, and management perspectives in water‐quality studies. The evidence synthesized in this review indicates that the principal challenge is no longer the absence of analytical techniques, but the limited comparability and integration of their outputs. Future methodological development should therefore prioritize five interconnected areas: reliable real‐time monitoring, integration of MP and nanoplastic characterization, interlaboratory standardization, multimodal analytical workflows, and data‐driven interpretation of high‐frequency observations. Progress in these areas would allow SPM monitoring to move from isolated concentration measurements toward integrated characterization of particle abundance, size, composition, contaminant association, and environmental behavior.

The selection of an optical or gravimetric method should be determined by the analytical objective, expected concentration range, required temporal resolution, and available resources. Gravimetric analysis is the most appropriate approach when a direct reference measurement of TSS is required, because it provides a mass‐based determination of the retained particulate fraction and is supported by standardized procedures [54, 55]. Turbidimetric and nephelometric methods are particularly suitable for routine monitoring of water turbidity, including automated measurements, with nephelometry being widely applied for quantitative turbidity determination [58, 59, 68, 69]. Laser‐ and IR‐based optical sensors are advantageous when high‐frequency or in situ measurements are required because of their rapid optical response and suitability for continuous monitoring; however, their calibration remains dependent on particle characteristics and the properties of the investigated water matrix [66, 67, 70]. The Secchi disk is more appropriate for lakes, reservoirs, and other open water bodies where rapid assessment of water transparency and long‐term spatial or temporal observations are required [71–73]. The Snellen cylinder provides a simple semiquantitative assessment of transparency in relatively small water samples and can therefore be useful for rapid laboratory or field comparison; however, its results are observer‐ and illumination‐dependent. In contrast, kaolin comparison scales represent a low‐cost visual approach that may be useful for preliminary classification when instrumental equipment is unavailable, although their results should be regarded as approximate and should be locally calibrated before conversion to instrumental units. Photometric measurements of transmitted light or OD are particularly useful under controlled laboratory conditions, especially when relative changes in particle concentration or water transparency are being investigated [60, 66]. Overall, instrumental methods are preferable when quantitative accuracy, reproducibility, or continuous monitoring is required, whereas visual methods remain useful for rapid surveys and resource‐limited applications. Overall, the analysis shows that the environmental role of SPM cannot be reduced to concentration metrics alone, as its behavior is governed by the coupled effects of particle size, composition, and ongoing transformation processes in the water column and at the sediment–water interface. The reviewed evidence consistently indicates that the same nominal SPM level may correspond to fundamentally different ecological conditions depending on the dominance of fine fractions, the presence of sorbed contaminants, and hydrodynamic context. This variability explains the limited transferability of empirical relationships and underlines the need for locally calibrated, process‐oriented interpretations. At the same time, the convergence of analytical approaches, from optical sensing to high‐resolution chemical methods, creates a basis for more integrated assessments, provided that their outputs are interpreted within a unified framework. In this context, the combined use of conceptual and multidimensional representations offers a practical way to relate measurements to mechanisms, improving the consistency of data interpretation and supporting more targeted decisions in monitoring and water management.

## 7. Conclusions

SPM is one of the main factors affecting water quality and ecosystem health, serving as both an indicator of anthropogenic pressure and a vector for the transport of pollutants. A wide range of methods is available for its detection and analysis, each with particular advantages and limitations. Gravimetric techniques provide robust quantitative data but are labor‐intensive, while optical instruments allow continuous measurements but require careful calibration to local conditions. Granulometric and chemical analyses reveal the interactions between particle size, composition, and contaminant binding, underlining the ecological significance of fine and chemically enriched fractions. Persistent problems include the lack of harmonized methodology, limited automation in field applications, and the growing challenge of monitoring MPs and nanomaterials. Future progress will depend on combining established approaches with advanced analytical and sensor technologies to achieve more reliable and comparable data. Such integration will support more accurate environmental assessments and more effective protection of water resources. Several methodological gaps remain. First, harmonization of operational definitions and fractionation procedures is needed to improve comparability among SPM datasets. Second, optical measurements require better site‐specific calibration and explicit reporting of measurement uncertainty. Third, particle‐size analysis remains sensitive to particle shape, aggregation, and optical‐property assumptions, requiring systematic validation against independent reference methods. Fourth, analytical workflows for MPs and nanoplastics require improved integration of particle‐size and polymer‐specific information. Finally, future monitoring should increasingly combine gravimetric, optical, particle‐sizing, and chemical measurements within integrated, locally calibrated frameworks.

## Funding

No funding was received for this manuscript.

## Conflicts of Interest

The authors declare no conflicts of interest.

## Data Availability

The data that support the findings of this study are available upon reasonable request to the corresponding author. Data used to support the findings of this study are included within the article.
